# Editorial: Advances in chronic pain treatment

**DOI:** 10.3389/fmed.2024.1496449

**Published:** 2024-10-04

**Authors:** Raúl Ferrer-Peña, Metoda Lipnik-Stangelj, Carlos Goicoechea

**Affiliations:** ^1^Department of Physiotherapy, Faculty of Health, La Salle University Center, Madrid, Spain; ^2^Clinico-Educational Research Group on Rehabilitation Sciences (INDOCLIN), CSEU La Salle, UAM, Madrid, Spain; ^3^Cognitive Neuroscience, Pain and Rehabilitation Research Group (NECODOR), Faculty of Health Sciences, Rey Juan Carlos University, Madrid, Spain; ^4^Institute of Pharmacology and Experimental Toxicology, Faculty of Medicine, University of Ljubljana, Ljubljana, Slovenia; ^5^Area of Pharmacology, Nutrition and Bromatology, Department of Basic Health Sciences, Universidad Rey Juan Carlos, Unidad Asociada de I+D+i al Instituto de Química Médica (IQM) CSIC-URJC, Alcorcón, Spain; ^6^High Performance Experimental Pharmacology Research Group, Universidad Rey Juan Carlos (PHARMAKOM), Alcorcón, Spain; ^7^Grupo Multidisciplinar de Investigación y Tratamiento del Dolor (i+DOL), Alcorcón, Spain

**Keywords:** chronic pain, neurophysiological mechanisms, risk factors, neuromodulation, personalized & precision medicine (PPM), biobehavioral, movement

## Introduction

Chronic pain is a multifaceted condition that affects millions globally (1), often persisting beyond 3 months and significantly impairing individuals' quality of life (2). In recent decades, significant advances have been made in understanding the pathophysiology of chronic pain, laying the groundwork for the development of new therapies and therapeutic approaches. This progress has been largely driven by advances in neuroscience, providing a deeper understanding of how the brain and central nervous system process pain (3). The development of neuroimaging techniques has allowed scientists to observe directly the alterations in brain activity associated with chronic pain, identifying abnormal patterns of neural activation and desynchronization that perpetuate pain perception (4, 5).

This growing understanding of chronic pain has led to the integration of multiple disciplines in its approach, from medicine and psychology to biotechnology and engineering (6). This multidisciplinary approach is crucial for addressing the complexity of this condition, enabling the development of strategies that not only focus on relieving pain but also address its underlying causes, thus optimizing long-term outcomes for patients (7).

## Neurophysiological mechanisms and risk factors

Understanding the neurophysiological mechanisms underlying chronic pain remains a priority within the field. Pinilla-Fernández et al. explore the neurobiological underpinnings of chronic neuropathic pain using advanced neuroimaging techniques. Where they quantified the difference in brain metabolite concentrations in whiplash-associated disorders (WAD) within the anterior cingulate and dorsolateral prefrontal cortex, and identified key metabolites associated with chronic neuropathic pain components during chronic WAD.

Additionally, Diao et al. employ a genetic approach to investigate modifiable risk factors in the development and progression of knee osteoarthritis. Through a Mendelian randomization study, they highlight the causal relationships between these risk factors and the disease, underscoring the importance of addressing modifiable elements in both preventive and therapeutic strategies. Together, these studies reinforce the need for a multifaceted approach to managing chronic pain, integrating neurophysiological insights with a focus on modifiable risk factors to optimize patient outcomes.

## Neuromodulation and pain management

One of the central themes in recent advances in chronic pain treatment is the role of neuromodulation in managing chronic pain conditions. Chang et al. provide a comprehensive review of the effectiveness of transcranial alternating current stimulation (tACS) in managing chronic pain. While results vary across different studies, this review highlights the potential of tACS as a non-invasive neuromodulatory tool, particularly for conditions resistant to conventional therapies. Moreover, Escobar-Sánchez et al. explore the long-term effects of high-frequency stimulation (HFS) on pain and sensitivity in healthy subjects. Their study illustrates significant bilateral changes in mechanical and thermal sensitivity after HFS, particularly in cold sensitivity, which persisted long after the stimulation ended. These findings suggest that demographic characteristics, such as age and gender, may influence the efficacy of neuromodulatory interventions. As research continues to explore pain mechanisms, these insights will be crucial for developing more targeted and effective therapies.

## Other innovative therapies and technology integration

The exploration of innovative therapies and technologies remains a critical focus in the current scientific landscape. Li et al. review the latest developments in biomimetic nanotechnology for drug delivery, particularly emphasizing its application in treating rheumatoid arthritis. They highlight the potential of biomimetic nano-systems to revolutionize the treatment of chronic inflammatory conditions by enhancing the precision and efficiency of drug delivery. This approach could significantly improve the management of diseases like rheumatoid arthritis, where targeted drug delivery is essential to improve therapeutic efficacy, minimize systemic side effects, and thus optimize patient outcomes.

Additionally, Doménech-García et al. investigate the complex interplay between placebo and nocebo effects in invasive treatments, such as percutaneous needle electrolysis and dry needling, for patients with patellar tendinopathy. Their research sheds light on the intricate psychological factors involved in chronic pain management, demonstrating how patient expectations can profoundly influence treatment outcomes.

## Personalized therapeutic approaches

The importance of personalized approaches in treating chronic pain is another significant theme within recent research. Escobar-Sánchez et al. highlight how different stimulation modalities can lead to variable changes in pain perception and sensitivity, emphasizing the necessity of tailoring pain management strategies to the specific characteristics of each patient. Similarly, Sreckovic et al. explore the role of peripheral nerve blocks in managing chronic post-surgical pain (CPSP) after knee arthroplasty. Their findings suggest that nerve blocks, such as the adductor canal block and interspace between the popliteal artery and capsule of the posterior knee block, significantly reduce opioid consumption and the incidence of CPSP, demonstrating that personalized regional analgesia can effectively improve postoperative outcomes and reduce long-term pain.

On the other hand, Del Piccolo et al. introduce the INTEGRO protocol, an innovative integrated psychotherapeutic intervention designed for fibromyalgia (FM) management. This protocol combines Cognitive Behavioral Therapy, Acceptance and Commitment Therapy, and somatic experiential techniques to address the complex, multifaceted nature of FM. Through a personalized approach that includes psychoeducation, emotion regulation, and self-efficacy enhancement, the INTEGRO protocol has demonstrated its effectiveness in reducing FM-related pain and improving the health-related quality of life in patients. Moreover, La Touche et al. introduce the BioPMovQ, a novel instrument designed to assess the relationship between pain and movement from a biobehavioral perspective. These studies collectively highlight the critical need for personalized therapeutic approaches in chronic pain treatment, emphasizing the role of individualized strategies in optimizing patient outcomes.

## Integrating clinical theory and practice

In addition to personalized treatments, it is also relevant to highlight the integration of theoretical knowledge into clinical practice advances. Zhai et al. revisit and refine the theory of trigger points and their role in musculoskeletal pain, offering a comprehensive review that emphasizes the importance of understanding muscle pain patterns in clinical practice considering biomechanics, kinesiology and compensatory mechanisms. Their work advocates for a more nuanced application of trigger point theory in clinical settings, emphasizing that a deeper grasp of these mechanisms can lead to improved patient outcomes.

## Conclusion

The articles featured in this analysis collectively advance our understanding of chronic pain and its management. By integrating neuromodulation, personalized therapies, theoretical insights, and innovative technologies, these studies offer a comprehensive overview of the current landscape in chronic pain treatment. The findings presented here not only deepen our understanding of chronic pain but also pave the way for future research and clinical applications that could significantly enhance patient care and outcomes.

## References

[1] Zimmer Z Fraser K Grol-Prokopczyk H Zajacova A. A global study of pain prevalence across 52 countries: examining the role of country-level contextual factors. Pain. (2022) 163:1740–50. 10.1097/j.pain.0000000000002557 35027516 PMC9198107

[2] Hadi MA McHugh GA Closs SJ. Impact of chronic pain on patients' quality of life: a comparative mixed-methods study. J Patient Exp. (2019) 6:133–41. 10.1177/2374373518786013 31218259 PMC6558939

[3] Chen ZS. Decoding pain from brain activity. J Neural Eng. (2021) 18:051002. 10.1088/1741-2552/AC28D4 34608868

[4] Bosma RL Hemington KS Davisa KD. Using magnetic resonance imaging to visualize the brain in chronic pain. Pain. (2017) 158:1192. 10.1097/j.pain.0000000000000941 28622273

[5] Martucci KT MacKey SC. Neuroimaging of pain: human evidence and clinical relevance of central nervous system processes and modulation. Anesthesiology. (2018) 128:1241–54. 10.1097/ALN.0000000000002137 29494401 PMC5953782

[6] Driscoll MA Kerns RD. Integrated, team-based chronic pain management: bridges from theory and research to high quality patient care. Adv Exp Med Biol. (2016) 904:131–47. 10.1007/978-94-017-7537-3_10 26900068

[7] Cohen SP Vase L Hooten WM. Chronic pain: an update on burden, best practices, and new advances. Lancet. (2021) 397:2082–97. 10.1016/S0140-6736(21)00393-7 34062143

